# Filaggrin-stratified transcriptomic analysis of pediatric skin identifies mechanistic pathways in patients with atopic dermatitis

**DOI:** 10.1016/j.jaci.2014.04.021

**Published:** 2014-07

**Authors:** Christian Cole, Karin Kroboth, Nicholas J. Schurch, Aileen Sandilands, Alexander Sherstnev, Grainne M. O'Regan, Rosemarie M. Watson, W.H. Irwin McLean, Geoffrey J. Barton, Alan D. Irvine, Sara J. Brown

**Affiliations:** a Division of Computational Biology, College of Life Sciences, University of Dundee, Dundee, United Kingdom; b Centre for Dermatology and Genetic Medicine, Division of Molecular Medicine, Colleges of Life Sciences and Medicine, Dentistry & Nursing, University of Dundee, Dundee, United Kingdom; c Department of Dermatology, Our Lady's Children's Hospital, Crumlin, Dublin, Ireland; d National Children's Research Centre, Our Lady's Children's Hospital, Crumlin, Dublin, Ireland; e Clinical Medicine, Trinity College Dublin, Dublin, Ireland

**Keywords:** Atopic dermatitis, direct RNA sequencing, eczema, filaggrin, gene expression, single molecule, skin, tissue, transcriptome, AD, Atopic dermatitis, *CILP*, Cartilage intermediate layer protein gene, DRS, Direct RNA sequencing, eQTL, Expression quantitative trait loci, FDR, False discovery rate, *FLG*, Filaggrin gene, GO, Gene ontology, STAT, Signal transducer and activator of transcription

## Abstract

**Background:**

Atopic dermatitis (AD; eczema) is characterized by a widespread abnormality in cutaneous barrier function and propensity to inflammation. Filaggrin is a multifunctional protein and plays a key role in skin barrier formation. Loss-of-function mutations in the gene encoding filaggrin *(FLG)* are a highly significant risk factor for atopic disease, but the molecular mechanisms leading to dermatitis remain unclear.

**Objective:**

We sought to interrogate tissue-specific variations in the expressed genome in the skin of children with AD and to investigate underlying pathomechanisms in atopic skin.

**Methods:**

We applied single-molecule direct RNA sequencing to analyze the whole transcriptome using minimal tissue samples. Uninvolved skin biopsy specimens from 26 pediatric patients with AD were compared with site-matched samples from 10 nonatopic teenage control subjects. Cases and control subjects were screened for *FLG* genotype to stratify the data set.

**Results:**

Two thousand four hundred thirty differentially expressed genes (false discovery rate, *P* < .05) were identified, of which 211 were significantly upregulated and 490 downregulated by greater than 2-fold. Gene ontology terms for “extracellular space” and “defense response” were enriched, whereas “lipid metabolic processes” were downregulated. The subset of *FLG* wild-type cases showed dysregulation of genes involved with lipid metabolism, whereas filaggrin haploinsufficiency affected global gene expression and was characterized by a type 1 interferon–mediated stress response.

**Conclusion:**

These analyses demonstrate the importance of extracellular space and lipid metabolism in atopic skin pathology independent of *FLG* genotype, whereas an aberrant defense response is seen in subjects with *FLG* mutations. Genotype stratification of the large data set has facilitated functional interpretation and might guide future therapy development.

Atopic dermatitis (AD; atopic eczema or eczema) is an itchy inflammatory skin disease with a spectrum of clinical skin phenotypes.^1,2^ The pathology of eczematous skin is characterized by epidermal intercellular edema and a barrier dysfunction resulting in increased transcutaneous water loss and increased penetration of external allergens and irritants.^3^ A subset of patients with AD show greater susceptibility to bacterial^4^ and viral^5^ infection.

Previous studies have investigated the transcriptome in atopic and eczematous skin by using microarray technologies and quantitative PCR, identifying a variety of pathomechanisms, including abnormalities in epidermal differentiation,^6-8^ inflammatory pathways,^9-14^ and lipid homeostasis.^12,15,16^ However, microarray analysis is intrinsically restricted by preselection of genes represented on an array and by annotations from which gene expression is quantified.^17^ Sequencing RNA is now an established methodology to study gene expression without the restriction of gene preselection.^18^ We chose to use single-molecule direct RNA sequencing (DRS)^19,20^ as the optimal methodology for quantification of the relatively small amounts of mRNA available from pediatric skin biopsy samples. DRS offers accurate quantification of polyadenylated RNA, avoiding bias that might be introduced by the steps of reverse transcription, ligation, and amplification in other forms of RNA sequencing. In addition, DRS provided information on the DNA strand from which the mRNA was transcribed; this facilitates more accurate alignment of reads to genes in the complex overlapping regions that are common in the human genome.^21^

The finding that loss-of-function mutations in the skin barrier gene encoding filaggrin *(FLG)* are strongly and significantly associated with AD risk^22,23^ has focused attention on skin barrier dysfunction as a primary pathogenic mechanism. Filaggrin deficiency appears to play a central role in the pathogenesis of AD. Filaggrin expression can be downregulated by T_H_2 cytokines^24-26^ and atopic inflammation,^27,28^ whereas *FLG* intragenic copy number variation influences AD risk in a dose-dependent fashion, even in the absence of *FLG* null mutations.^29^ Filaggrin is expressed initially as a long insoluble polyprotein, profilaggrin, which is cleaved to produce functional monomers that aggregate and align keratin filaments.^30^ Filaggrin is thereafter degraded in a multistep proteolysis to release hygroscopic amino acids, contributing to the skin's “natural moisturizing factor.”^31,32^ The mechanisms by which the multifunctional profilaggrin/filaggrin proteins lead to AD, as well as atopic asthma, allergic rhinitis, food sensitization, and peanut allergy, have been the focus of intense study over recent years.^33^

We sought to undertake a comprehensive analysis of the primary molecular abnormalities in atopic skin using accurate quantification of the transcriptome in pediatric patients with AD compared with nonatopic teenage control subjects. *FLG* genotype was used to define subphenotypes for the stratification of this large data set. Uninvolved skin was chosen to study the underlying pathomechanisms of AD without the confounding effects of secondary inflammation or infection. Our strategy for analysis was stepwise, as follows: (1) all cases compared with all control subjects, (2) case-control comparison stratified by *FLG* genotype, and (3) case-case comparison stratified by *FLG* genotype (Fig 1).

## Methods

### Study subjects: Patients with AD

This study was reviewed and approved by the research ethics board at Our Lady's Children's Hospital, Dublin, Ireland (reference: SAC/119/09 26). Pediatric patients with AD of Irish ethnicity attending the dermatology department were invited to participate. Twenty-six children aged 6 to 16 years and their parents/guardians provided written informed consent; 19 were male, and 7 were female (Table I). Each child had a history of chronic relapsing AD diagnosed by experienced pediatric dermatologists (G.M.O'R., R.M.W., A.D.I., and S.J.B.) and moderate or severe disease, as defined according to the Nottingham Eczema Severity Score.^34^ Children with a history of current or previous treatment with systemic immunomodulatory medication were excluded. The area of biopsy was not treated with topical steroids for 4 weeks before sampling, and emollients were not applied for 2 days before biopsy. A single punch biopsy specimen of 3 to 4 mm in diameter was taken from each child after local anesthetic injection and standard aseptic technique from the upper outer buttock skin. Clinically uninvolved skin was sampled and protected skin on the buttock was chosen to minimize differences resulting from UV exposure and variation in environmental humidity.^35^ Batch effects and RNA degradation were minimized because samples were collected by 2 clinicians (G.M.O'R. and S.J.B.), and the biopsy specimens were immediately snap-frozen in liquid nitrogen before storage in a single −80°C freezer before simultaneous processing. The study subjects had previously provided blood samples for DNA extraction as part of the ongoing National Children's Research Centre AD Case Collection.^36^

### Study subjects: Nonatopic control subjects

The collection of samples from healthy volunteers was reviewed and approved by the East of Scotland Research Ethics Service, United Kingdom (LR/11/ES/0043). Nonatopic teenage volunteers with 4 grandparents of Irish or Scottish descent were recruited. Skin biopsy specimens were collected in the same way as for the patients with pediatric AD, and 5 mL of venous blood was collected for DNA extraction.

### RNA extraction and quality control

The protocol for extraction of total RNA of greater than 200 nucleotides in length from tissue by using the Qiagen RNeasy Mini Kit (Qiagen, Manchester, United Kingdom) was modified and optimized, as follows. Working on dry ice, subcutaneous fat was trimmed from the skin biopsy specimen, and the remaining sample was placed in a 2-mL sample tube RB (Qiagen, UK) with 600 μL of Buffer RLT (Qiagen, UK) and one 7-mm stainless steel bead (Qiagen, UK). The sample was disrupted and homogenized at 4°C in a Qiagen TissueLyser LT for 5 minutes at 50 oscillations per second. RNA extraction then proceeded according to a standard protocol with 2 final elution steps each using 30 μL of RNase-free water and centrifuged at 12,000 rpm for 90 seconds. Spectrophotometry (Nano-Drop ND-1000 spectrophotometer; Thermo Scientific, Uppsala, Sweden) and a microfluidics platform for size analysis and quantification (Agilent 2100 Bioanalyser; Agilent Technologies, Santa Clara, Calif) were used for quality control. This protocol yielded 2.5 to 10.8 μg per sample of RNA with an *A*_260/280_ ratio of 1.9 to 2.1.

### *FLG* genotyping

Genomic DNA samples extracted from blood were initially screened for *FLG* null mutations with TaqMan allelic discrimination assays (TaqMan, Applied Biosystems 7700 sequence detection system; Applied Biosystems, Foster City, Calif), as previously described.^37^ Next-generation deep sequencing of 25 cases and all 10 control subjects identified additional mutations that were confirmed by using Sanger sequencing.

### Measurement of global transcript abundance

DRS was performed on a HeliScope Sequencer (Helicos, Cambridge, Mass) with 500 ng of extracted RNA, as previously described.^19^ RNA molecules are captured by the poly-A tail, and the single molecule of mRNA is directly sequenced from the 3′ end, producing reads up to 70 bp in length (median, 32 bp).

### DRS read processing

Details of sequence alignment and analysis are presented in the Methods section in this article's Online Repository at www.jacionline.org. DRS resulted in 480 million reads across all 36 samples, providing gene expression data for 11,259 genes. The raw data are deposited at the European Genome-Phenome Archive (EGAS00001000823/EGAC00001000200); processed data are available at polyAdb (http://www.compbio.dundee.ac.uk/polyAdb) and can be viewed in the Integrated Genome Browser or as data tracks at www.ensembl.org. Scripts for performing analysis and generating the figures that accompany this article are available at polyAdb (http://www.compbio.dundee.ac.uk/polyAdb).

### Differential gene expression and *FLG* correlation analyses

EdgeR (version 2.6.12 in R version 2.15.1) analysis was performed with generalized linear models to control for sex bias between comparisons. Unless otherwise stated, genes were called as significantly differentially expressed if the Benjamini-Hochberg corrected false discovery rate (FDR) was less than 0.05.^38^ All-against-all correlation of gene expression across individual samples was performed with the Pearson method. Further details are presented in the Methods section in this article's Online Repository.

### Gene ontology analysis and functional protein network analysis

Gene ontology (GO) has been developed to provide a controlled vocabulary of terms to describe the characteristics of genes and gene products with standardization across species and between databases. This allows the bioinformatic analysis of GO terms in large data sets for thematic classification. GO analyses were performed with AmiGO gene ontology version 1.8 (http://amigo.geneontology.org/cgi-bin/amigo/search.cgi?action=advanced_query). Functional protein association networks were investigated *in silico* by using STRING_9.05_ (http://string-db.org/).

### Quantitative PCR analysis

RNA (1.4 ng) was converted to cDNA by using the High Capacity cDNA Reverse Transcription Kit (Applied Biosystems), according to a standard protocol. Aliquots of RNA extracted from atopic skin samples were analyzed in triplicate by using real-time quantitative PCR performed according to standard protocols with the TaqMan 7900HT Fast (Applied Biosystems) with normalization to glyceraldehyde-3-phosphate dehydrogenase *(GAPDH)*.

## Results

### Seventy-three percent of patients with AD carry 1 or more *FLG* null mutations

Our comprehensive screen for *FLG* null mutations revealed a high proportion of mutation carriers (19/26 [73%] of cases, Table I), reflecting the severity of disease and enrichment for coexisting ichthyotic skin phenotype. One heterozygous mutation, p.Y2092X, is reported for the first time. Two (20%) of 10 control subjects were heterozygous for an *FLG* null mutation (Table I).

### *FLG* genotype affects global gene expression

**Fig 2:**
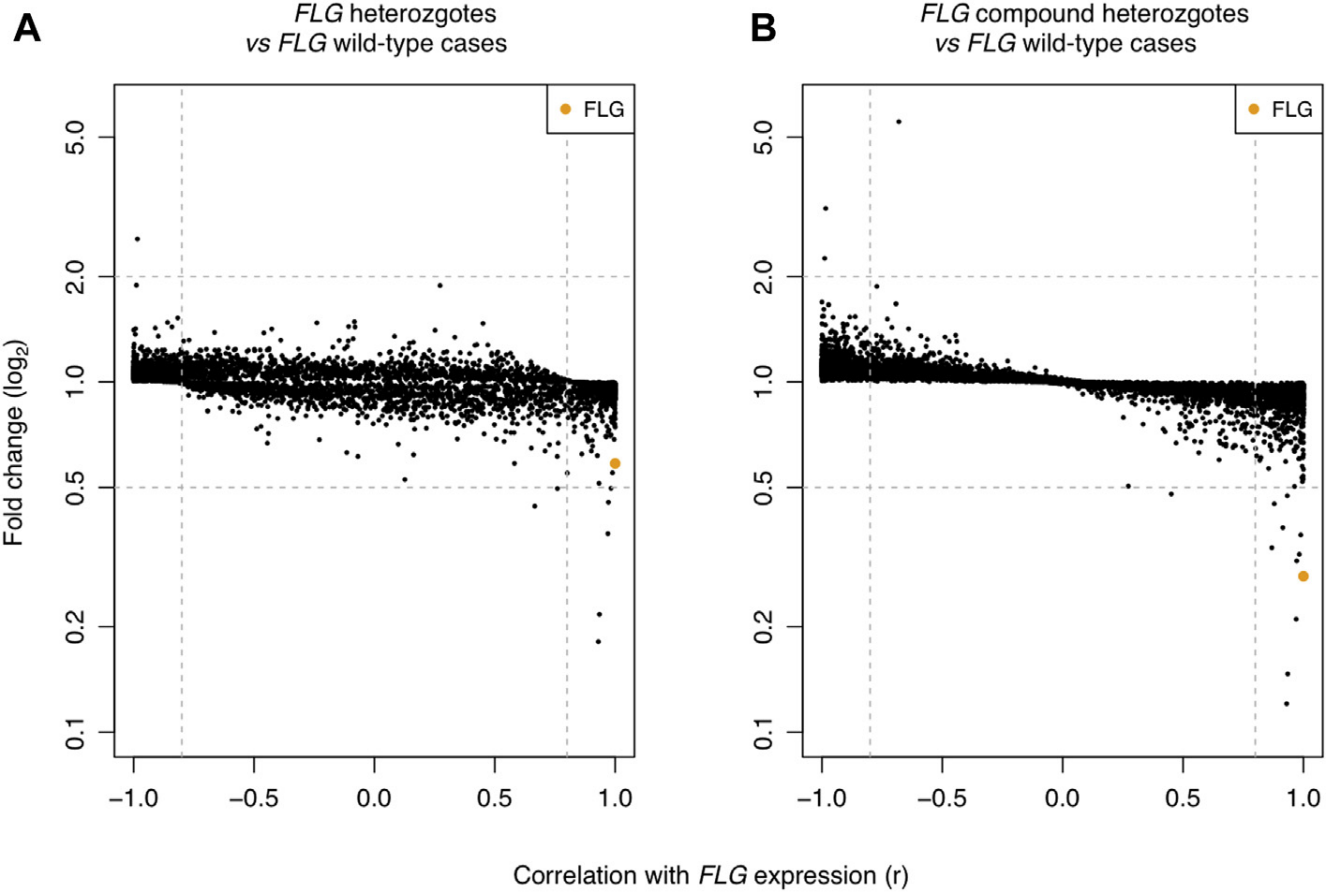
Scatterplots showing correlation of gene expression levels with *FLG* expression. The fold change of significantly differentially expressed genes (FDR *P* < .05) is plotted against the Pearson correlation coefficient for correlation with *FLG* expression. Each point represents expression of a single gene. *FLG* is marked in *orange*. **A,** Correlation of *FLG* wild-type (n = 7) compared with *FLG* heterozygous (n = 12) samples. **B,** Correlation of *FLG* wild-type (n = 7) versus *FLG* compound heterozygous (n = 7) samples.

Gene expression between samples was highly correlated (Pearson *r* = 0.81-0.99, see Fig E1 in this article's Online Repository at www.jacionline.org), and comparison of the control samples with AD cases revealed no obvious clustering by phenotype or *FLG* genotype (see Fig E2 in this article's Online Repository at www.jacionline.org). This highlights the subtlety of changes in gene expression in uninvolved atopic skin. The samples do not cluster according to age, providing some assurance that the use of samples from older subjects in the control group does not result in bias to account for the observed differences in transcriptional profile. However, in contrast, correlation with *FLG* gene expression showed a striking global change between samples of different *FLG* genotypes (Fig 2).

### “Defence response” and “extracellular region” genes are overexpressed in atopic skin, whilst “lipid metabolic processes” and “small molecule metabolic processes” are downregulated

Comparing all 26 cases with all 10 control subjects showed 2430 differentially expressed genes with an FDR of less than 0.05, including 211 with a fold change of greater than 2.0 and 490 genes with a fold change of less than 0.5. The full list is shown in Table E1 in this article's Online Repository at www.jacionline.org. GO analysis identified the most highly significant terms in the greater than 2.0-fold upregulated genes as “defense response” (28 genes, FDR: *P* = 7.1 × 10^−07^), the “extracellular region” or “extracellular space” (each 32 genes, FDR: *P* = 1.8 × 10^−3^) and “receptor binding” (23 genes, FDR: *P* = 5.7 × 10^−3^). The most highly significant terms in the less than 0.5-fold downregulated genes were “lipid metabolic process” (52 genes, FDR: *P* = 6.6 × 10^−11^), “small molecule metabolic process” (76 genes, FDR: *P* = 6.6 × 10^−11^), and “organic acid metabolic process” (44 genes, FDR: *P* = 6.6 × 10^−11^). The full list is shown in Table E2 in this article's Online Repository at www.jacionline.org.

**Fig E3:**
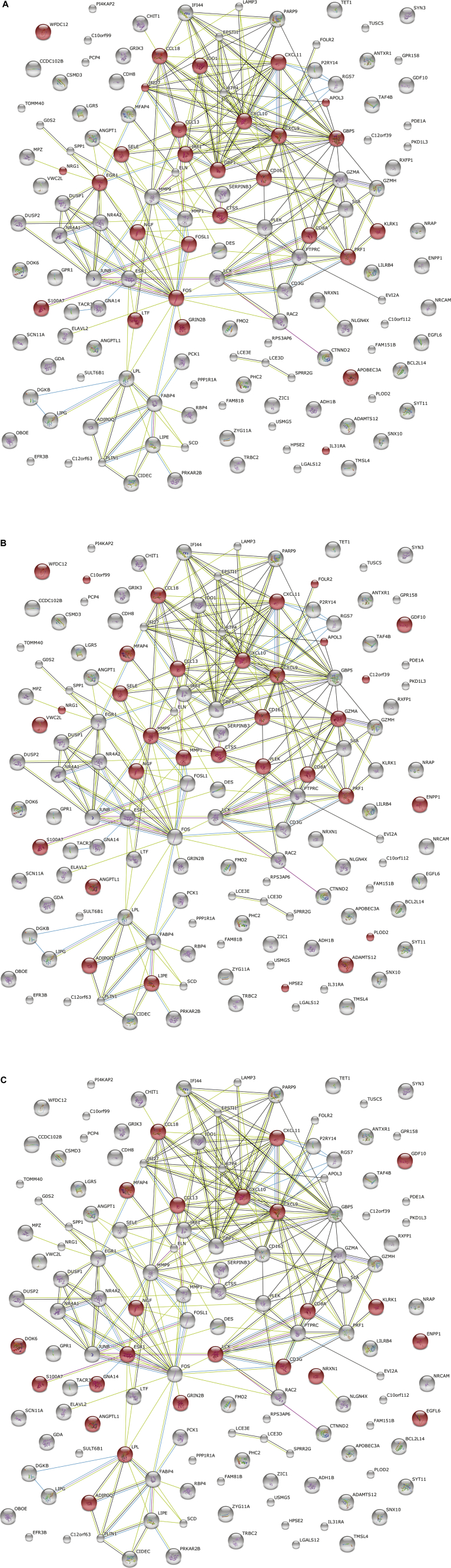


**Fig E4:**
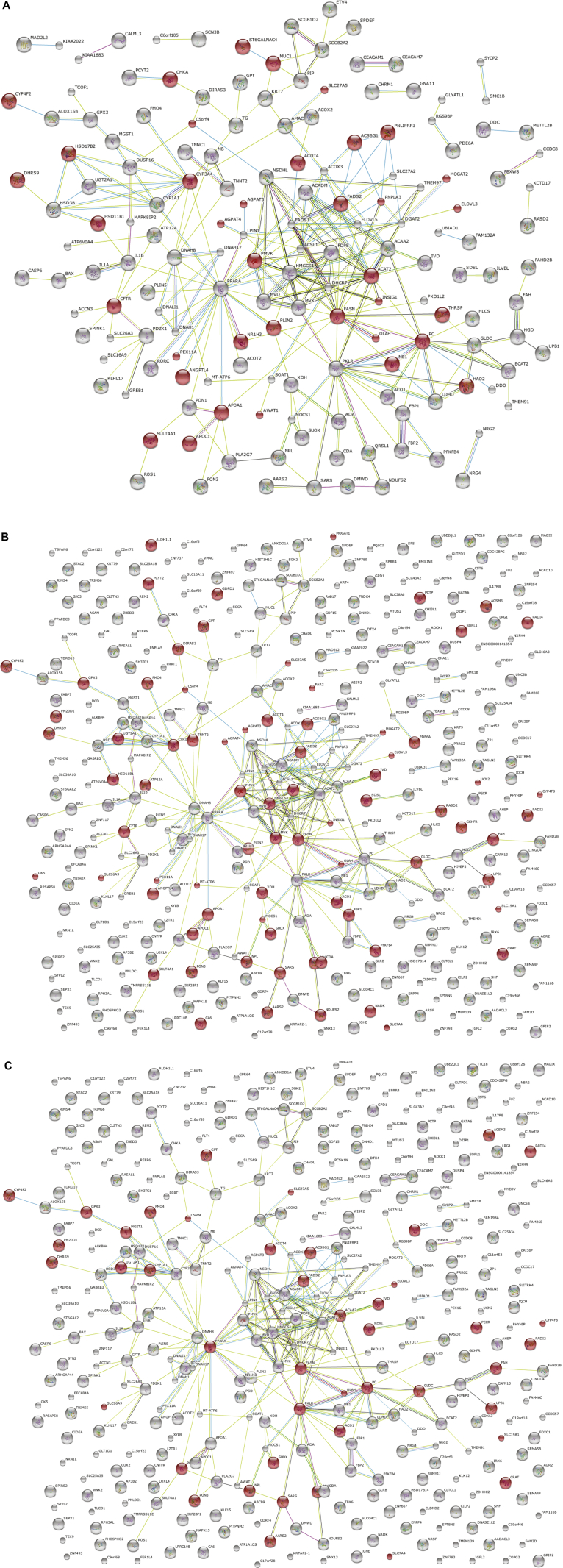


Analysis of the significantly upregulated genes identified predicted protein interaction networks classified as “defense response,” “extracellular region,” and “receptor binding” (see Fig E3 in this article's Online Repository at www.jacionline.org). Significantly downregulated genes show predicted functional networks within “lipid metabolic processes,” “small molecule metabolism,” and “organic acid metabolic processes” (see Fig E4 in this article's Online Repository at www.jacionline.org).

### *FLG* wild-type subjects show dysregulation of genes involved with lipid metabolism and upregulation of extracellular matrix terms

**Fig 3:**
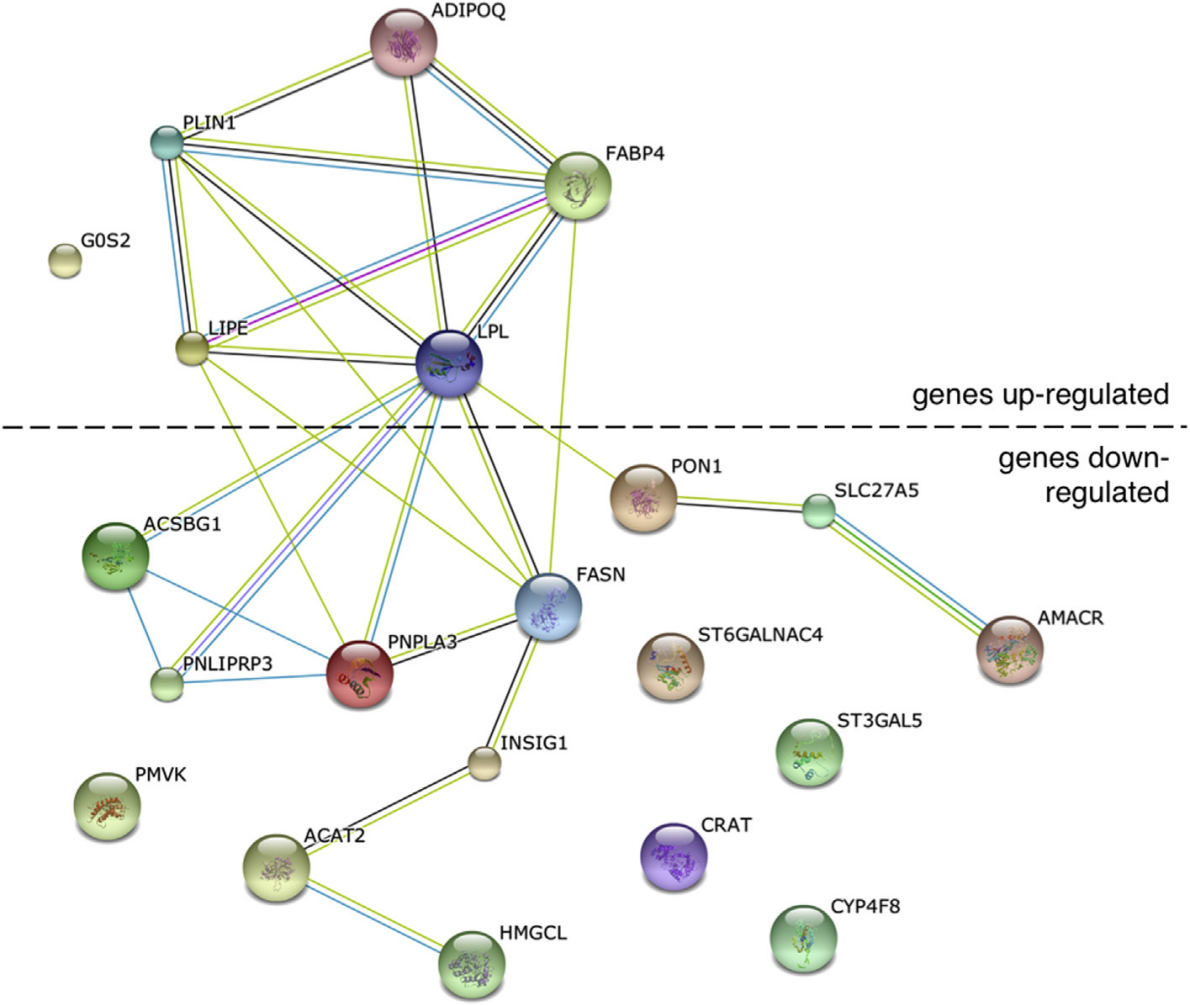
Network analysis of lipid metabolism genes differentially expressed in *FLG* wild-type AD cases compared with *FLG* wild-type control subjects. Twenty-one significantly differentially expressed genes (defined as FDR: *P* < .05 and fold change >2.0 or <0.05) were classified with the GO terms “lipid metabolic process” (n = 13), “cellular lipid metabolism” (n = 13), “lipid particle” (n = 4), and “triglyceride catabolism” (n = 3). Linear connectors indicate evidence for association in published data sets (STRING_9.05_; accessed March 3, 2014).

Analysis of the 8 *FLG* wild-type control subjects compared with the 7 *FLG* wild-type cases identified 401 differentially expressed genes with an FDR of less than 0.05, including 105 with a fold change of greater than 2.0 and 87 with a fold change of less than 0.5 (the full list can be found in Table E3 in this article's Online Repository at www.jacionline.org). Significant GO terms in the transcripts upregulated more than 2.0-fold included “extracellular region” (17 genes, FDR: *P* = 3.0 × 10^−3^) and “lipid particle” (4 genes, FDR: *P* = 3.0 × 10^−3^). Significantly downregulated transcripts (fold change <0.5) were classified with the GO terms “cellular lipid metabolic process” (13 genes, FDR: *P* = 1.6 × 10^−3^) and “lipid metabolic process” (13 genes, FDR: *P* = 2.7 × 10^−2^; the full list can be found in Table E2). *In silico* protein network analysis of the differentially expressed lipid metabolism genes shows a predicted network involving both upregulated and downregulated transcripts (Fig 3).

### Case-control analysis stratified by *FLG* genotype shows upregulated defense response cytokines and downregulated “steroid metabolic process”

**Fig E5:**
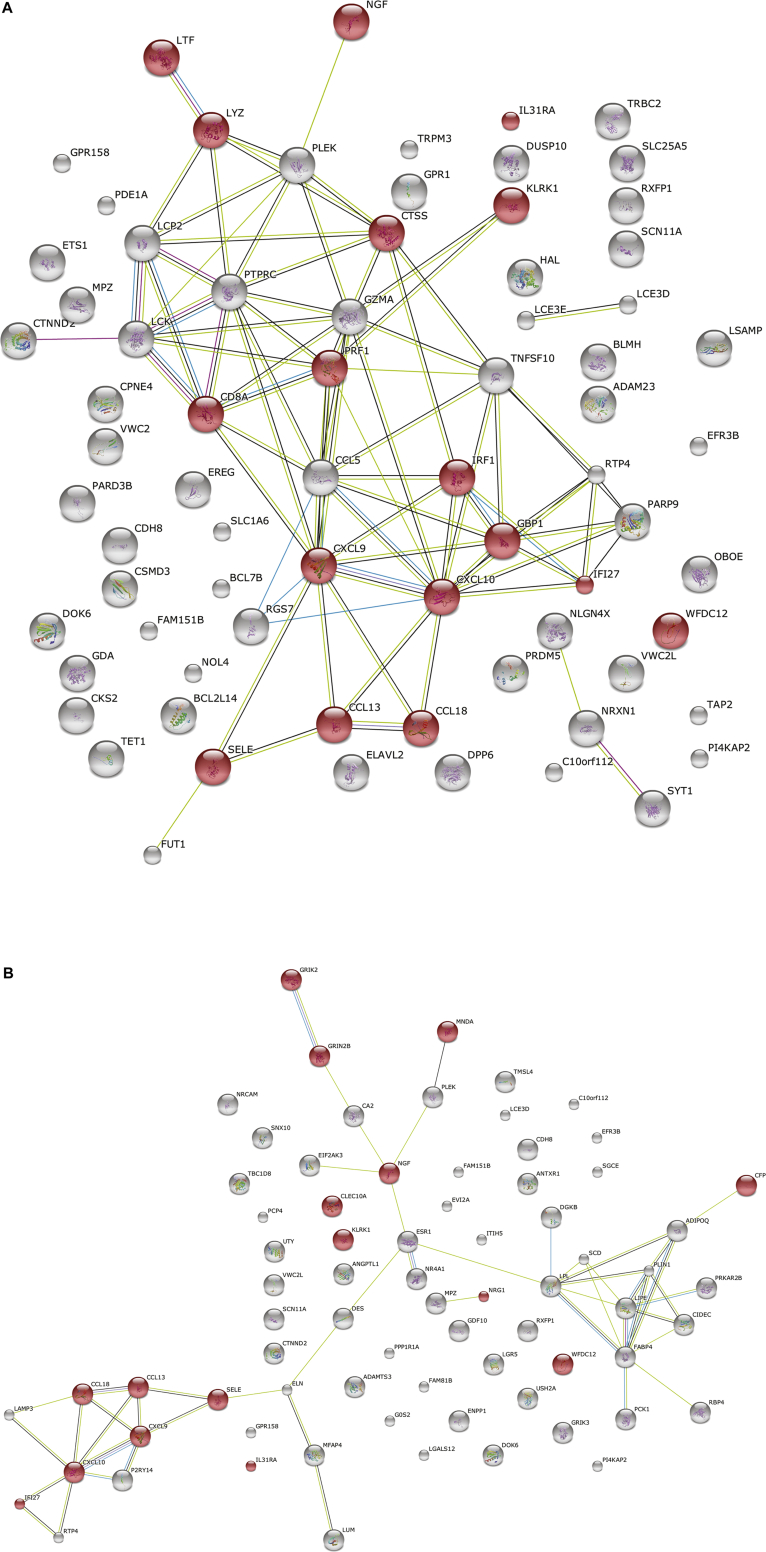


Analysis stratified according to *FLG* genotype was performed to investigate the mechanisms by which filaggrin haploinsufficiency might predispose to the development of AD. The 8 *FLG* wild-type control subjects were compared with the 7 compound heterozygous cases, revealing 816 differentially expressed transcripts (FDR <0.05). These included 137 with a fold change of greater than 2.0 and 266 with a fold change of less than 0.5 (the full list can be found in Table E4 in this article's Online Repository at www.jacionline.org). Significant GO terms in the upregulated transcripts included “defense response” (17 genes, FDR: *P* = 2.3 × 10^−4^) and “response to biotic stimulus” (14 genes, FDR: *P* = 2.3 × 10^−4^), “chemokine activity” (5 genes, FDR: *P* = 1.3 × 10^−3^), and “chemokine receptor binding” (5 genes, FDR: *P* = 1.3 × 10^−3^; full list shown Table E2). *In silico* protein network analysis of the upregulated transcripts demonstrated a functional network of chemokines and cytokines in the defense response, including both T_H_1- and T_H_2-associated transcripts: *CXCL9*, *CXCL10*, *CCL13*, *CCL18*, *SELE*, *IFI27*, and *IRF1* (see Fig E5, *A*, in this article's Online Repository at www.jacionline.org). The most significant GO term in the transcripts downregulated less than 0.5-fold was “steroid metabolic process” (14 genes, FDR: *P* = 3.6 × 10^−3^, see Table E2).

Analysis of the 8 *FLG* wild-type control subjects compared with 12 *FLG* heterozygous cases revealed 1139 differentially expressed transcripts (FDR, <0.05) in which 104 are significantly upregulated (fold change, >2.0) and 313 are significantly downregulated (fold change, <0.5; the full list can be found in Table E5 in this article's Online Repository at www.jacionline.org). The upregulated transcripts showed enrichment for GO terms in the “defense response” (16 genes, FDR: *P* = 7.6 × 10^−3^) forming a predicted protein network analogous to that seen in the wild-type control subject versus compound heterozygous case comparison, with the addition of *GRIN2B*, *GRIK2*, and *MNDA* (see Fig E5, *B*). Genes classified for the ontology terms “receptor binding” (15 genes, FDR: *P* = 2.9 × 10^−12^), “cytokine activity” (7 genes, FDR: *P* = 2.9 × 10^−12^), and “extracellular region” (19 genes, FDR: *P* = 3.2 × 10^−6^) were also significantly upregulated. The most highly significant GO term in the transcripts downregulated less than 0.5-fold were “small molecule metabolic process” (60 genes, FDR: *P* = 3.1 × 10^−10^), “carboxylic acid metabolic process,” and “oxoacid metabolic process” (each 35 genes and FDR: *P* = 3.1 × 10^−10^; the full list can be found in Table E2).

### *FLG* mRNA shows a stepwise reduction in patients with AD, and there might be upregulation of expression in *FLG* wild-type atopic skin

**Fig 4:**
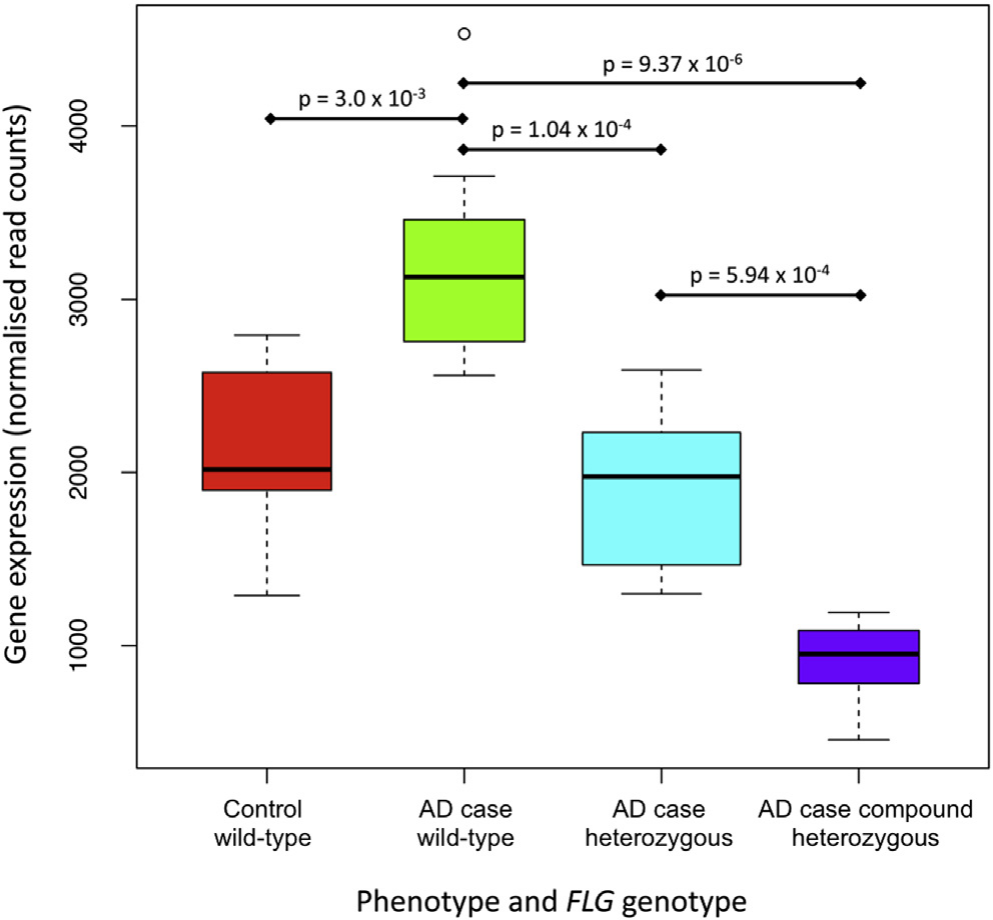
Box plot showing *FLG* mRNA read counts in different AD phenotypes and *FLG* genotypes. Gene expression was normalized across all samples with EdgeR: 8 wild-type control subjects, 7 wild-type AD cases, 12 heterozygous cases, and 7 compound heterozygous cases.

*FLG* mRNA–normalized read counts show a stepwise reduction from *FLG* wild-type to *FLG* heterozygous and compound heterozygous patients with AD (Fig 4). This indicates that a form of nonsense-mediated decay occurs in the context of an *FLG* null mutation, although mature mRNA transcripts are still detectable even in those subjects with 2 *FLG* null mutations (Fig 4). Comparison of all cases with all control subjects showed no significant difference in *FLG* mRNA levels (*P* > .05), but there is significantly greater mRNA expression in wild-type AD cases than wild-type control subjects (*P* = 3.0 × 10^−3^), suggesting that there might be a compensatory upregulation of *FLG* mRNA in atopic skin of *FLG* wild-type subjects.

### Differentially expressed transcripts at loci near to regions identified by genome-wide association studies indicate expression quantitative trait loci

Significantly differentially expressed transcripts in the case-control analysis were compared with AD-associated single nucleotide polymorphisms from published genome-wide association studies (see the Methods section in this article's Online Repository). Seventeen putative expression quantitative trait loci (*cis*-eQTLs) were identified on the basis of a transcript proximity of less than 250 kb between the single nucleotide polymorphism site and the 5′ end of the transcript (see Table E6 in this article's Online Repository at www.jacionline.org).^39^ Four of the proposed *cis*-eQTLs correspond to previously reported AD candidate genes (*FLG*, *TNXB*, *C11ORF30*, and *ZNF652*), whereas 13 represent novel candidates (see Table E6).

### Analysis of cases stratified by *FLG* genotype shows differential expression of cartilage intermediate layer protein *(CILP)*

A comparison of the 26 cases stratified by *FLG* genotype was performed to investigate filaggrin-associated mechanisms in AD pathogenesis; this comparison also represents the most closely matched samples to control for age-specific differences. A total of 201 genes were differentially expressed (unadjusted *P* < .01): 87 genes were differentially expressed in the *FLG* wild-type cases versus compound heterozygous cases, and 127 were differentially expressed in the wild-type versus heterozygote comparison (see Table E7 in this article's Online Repository at www.jacionline.org). Forty-one genes showed a fold change of greater than 2.0 or less than 0.5. After controlling for multiple testing, 2 genes showed a statistically significant difference in expression: *FLG* (FDR: *P* = 6.1 × 10^−12^; fold change, 0.3) and cartilage intermediate layer protein (*CILP*; FDR: *P* = .03; fold change, 0.2). The differential expression of *FLG*, *CILP*, and selected other transcripts was validated by using quantitative PCR with aliquots of the previously extracted RNA samples (see Table E8 in this article's Online Repository at www.jacionline.org).

GO analysis of the 127 transcripts showing differential expression between *FLG* wild-type and compound heterozygous cases showed the highest number of genes to be associated with “extracellular region” (28 genes, including *FLG* and *CILP*; FDR: *P* = 8.7 × 10^−3^), “carbohydrate binding” (12 genes, FDR: *P* = 5.6 × 10^−3^), and “calcium ion binding” (14 genes, including *FLG*; FDR: *P* = .02; the full list can be found in Table E9 in this article's Online Repository at www.jacionline.org).

### *FLG* expression correlates with gene expression in the extracellular space and is anticorrelated with a network of defense response genes

Correlation of gene expression with *FLG* expression was used to investigate filaggrin-related mechanisms and pathways in atopic skin. Twenty genes show strong correlation with *FLG* expression (each *r* > 0.98 and *P* < .05), including 7 classified within the extracellular region: *CA2* (carbonic anhydrase 2), *COL12A1* (collagen, type XII, alpha 1), *MUCL1* (mucin-like 1), *PIP* (prolactin-induced protein), *PRELP* (proline/arginine-rich and leucine-rich repeat protein), *SCGB1D2* (secretoglobin, family 1D, member 2), and *ZG16B* (zymogen granule protein 16 homolog B; the full list can be found in Table E10 in this article's Online Repository at www.jacionline.org).

**Fig 5:**
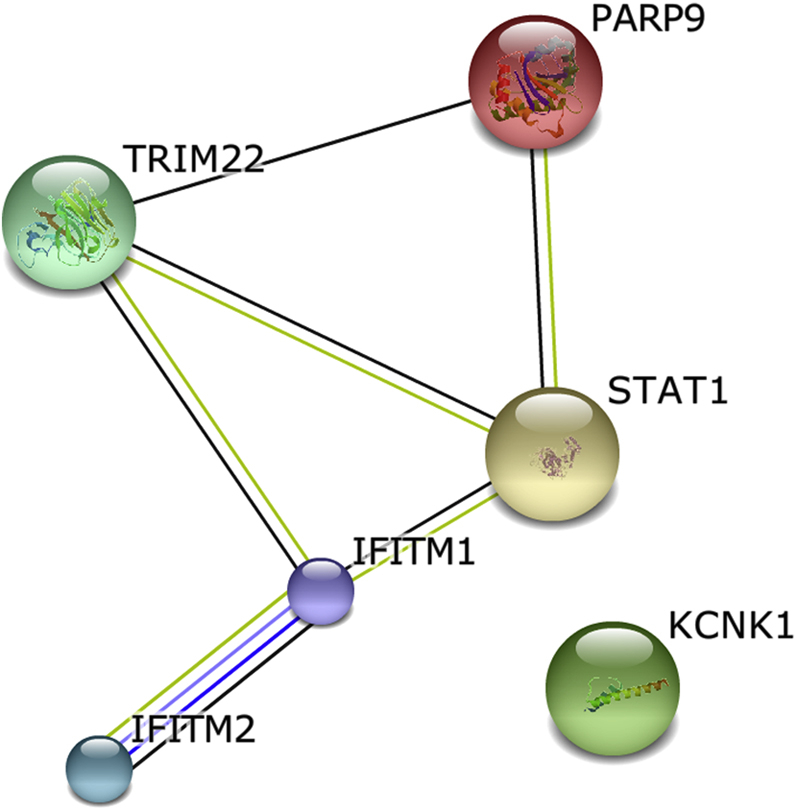
Network analysis of 6 genes with expression strongly anticorrelated with *FLG* expression: *TRIM22* (tripartite motif containing 22), *KCNK1* (potassium channel, subfamily K, member 1), *PARP9* (poly (ADP-ribose) polymerase family, member 9), *IFITM2* (interferon induced transmembrane protein 2), *IFITM1* (interferon induced transmembrane protein 1), and *STAT1* (STRING_9.05_; accessed October 27, 2013).

**Fig 6:**
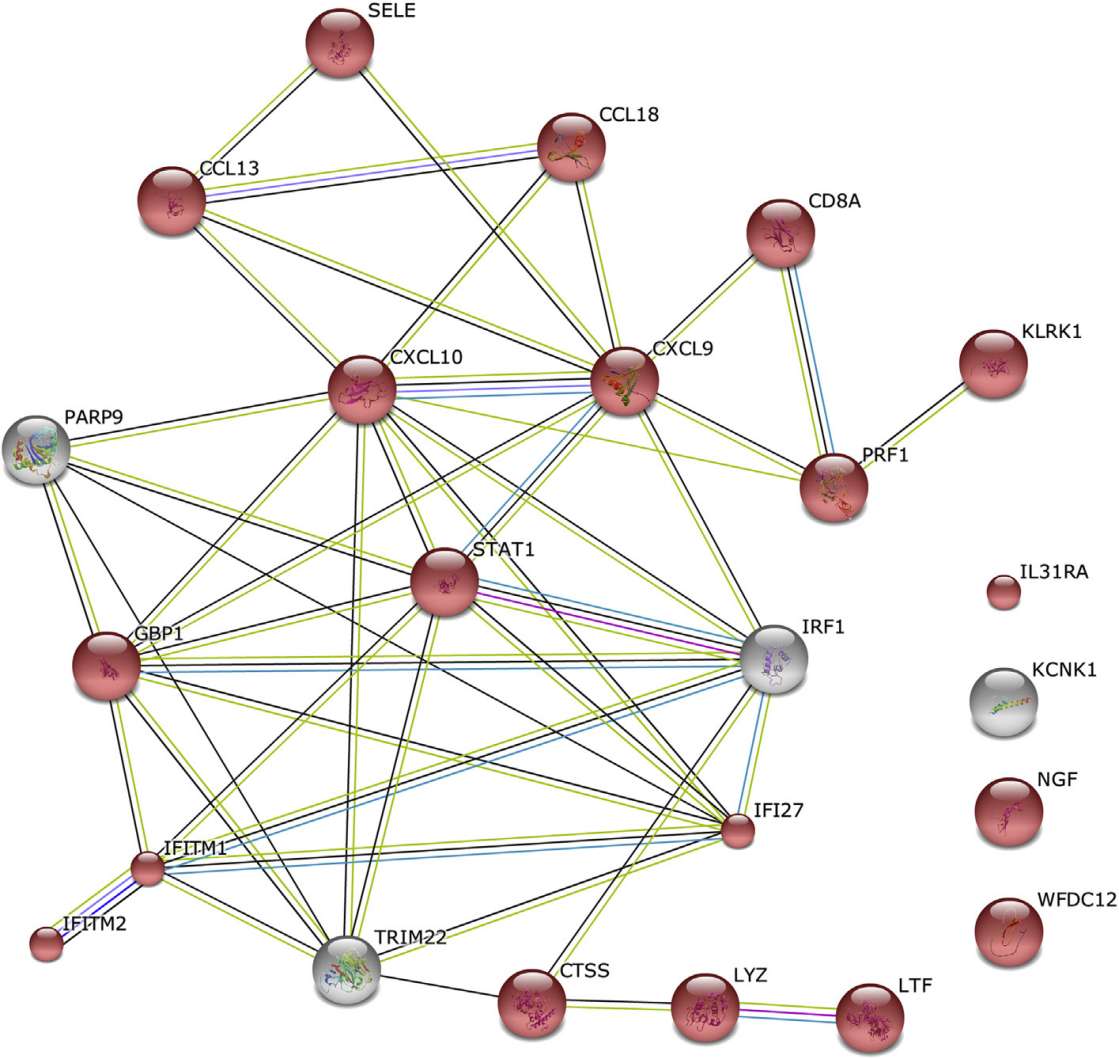
Genes anticorrelated with *FLG* and upregulated in *FLG* mutant cases show a common network of stress response. Six genes are anticorrelated (upregulated) with *FLG* expression, and 17 genes are upregulated in the *FLG* genotype–stratified case-control analyses. Proteins encoded by genes classified with the GO term “response to stress” are colored red (STRING_9.05_; accessed November 16, 2013).

The expression levels of 6 genes were strongly anticorrelated with *FLG* expression (each *r* < −0.98, see Table E10); 5 of these 6 genes formed a predicted network (Fig 5). Significant GO terms associated with this network include “response to virus” (4 genes, FDR: *P* = 5.1 × 10^−3^), “cellular response to type I interferon,” “response to type I interferon,” and “type I interferon–mediated signaling pathway” (each 3 genes, FDR: *P* = 6.3 × 10^−3^). Combining these 6 anticorrelated and upregulated genes with the 17 defense response genes that are upregulated in the *FLG* genotype–stratified case-control analyses (see Table E2) shows a common predicted functional network of “defense response” (20 genes, FDR: 5.6 × 10^−18^; Fig 6).

## Discussion

The identification of genes involved in the pathogenesis of AD represents a significant challenge because of the clinical heterogeneity and complexity of multiple interrelated genetic and environmental mechanisms in patients with this disease. The identification of null mutations within the gene encoding filaggrin *(FLG)* as a strong and significant risk factor for AD^22^ represented a fundamental breakthrough in understanding pathogenesis.^33^ The strong effect of filaggrin haploinsufficiency can be used to define AD subphenotypes clinically,^40^ and we have applied this insight for stratification of the large and complex data set generated by using transcriptomic analysis.

This study used DRS to quantify the whole transcriptome of atopic skin in a unique collection of pediatric AD skin biopsy specimens; it represents the largest collection of AD skin transcriptomes reported to date. Skin offers the advantage of sampling the tissue of interest, maximizing power to detect expression traits correlating with clinical phenotype.^41^ Importantly, the histopathology of clinically uninvolved atopic skin demonstrates an absence of inflammatory cell infiltrate, and gene expression changes are therefore likely to represent keratinocyte-related mechanisms rather than those from any other cell type. The skin of patients with AD shows epidermal barrier dysfunction, which can be demonstrated in nonlesional (clinically uninvolved) skin, as well as areas of active eczema.^42,43^ Nonlesional skin was sampled to focus on the intrinsic biological abnormality in atopic skin and to exclude, as far as possible, the secondary effects of inflammation in patients with active dermatitis and secondary infection, which would confound the mRNA profile.^6,10^ The cutaneous gene expression profile is known to vary by age, sex, and, most significantly, body site.^44^ This study included case-case comparison for optimal matching in age and skin site, and our case-control samples were carefully matched for body site and sex. Age matching in the case-control analysis was limited by the availability of skin biopsy samples from healthy children; however, the data obtained from biopsy specimens of teenage volunteers showed no clustering by age (see Fig E2). Furthermore, the differentially expressed defense response genes formed an overlapping network with the age-matched case-case analyses. DRS allows accurate quantification of mRNA species. The confirmation of findings from previous microarray studies and consistency with our own quantitative PCR studies provide support for the validity of DRS as a novel technique for the investigation of AD pathogenesis.

We have shown that genes encoding proteins in the extracellular space are differentially expressed in atopic skin, with upregulation in patients with AD compared with that seen in control subjects (see Table E2). Conversely, 7 genes encoding proteins in the extracellular region are downregulated in strong correlation with *FLG* expression (see Table E10). These genes are likely to contribute to mechanisms by which a quantitative reduction in intracellular filaggrin levels results in the paracellular barrier defect that is observed *in vitro*.^45,46^ Expression of *CILP* shows the most significantly reduced expression in *FLG* null cases compared with *FLG* wild-type cases and might represent a novel AD candidate gene. The protein encoded by *CILP* is expressed in many tissues, including skin and blood, as well as articular cartilage. It is secreted into the extracellular space and sequesters growth factors, cytokines, and matrix metalloproteases in the extracellular matrix. Also, it has been shown to antagonize the actions of TGF-β1 and insulin-like growth factor 1.^47,48^ We hypothesize that a reduction in expression of the cartilage intermediate layer protein permits increased activity of insulin-like growth factor 1 and TGF-β1, leading to cellular proliferation, whereas a reduction in the sequestration of proinflammatory cytokines and metalloproteases in the extracellular space might simultaneously contribute to skin barrier dysfunction in cases of AD associated with filaggrin deficiency.

The second major finding of this global transcriptomic analysis is the dysregulation of lipid metabolic pathways both in the unstratified case-control comparison (see Fig E4, *A*) and in the *FLG* wild-type case-control comparison (Fig 3). The demonstration of lipid dysregulation predominantly in the cases without *FLG* mutations is in keeping with *in vivo*^49^ and *in vitro*^50^ findings that filaggrin deficiency does not affect lipid composition in the stratum corneum. Previous microarray analyses have shown a reduction in expression of lipid homeostatic genes^12^ and reduced intercellular lipid levels.^15^ Organotypic culture of primary keratinocytes has shown increased expression of a cluster of genes associated with lipid metabolism throughout differentiation in parallel with increasing barrier properties.^51^ Lipid raft disruption produces transcriptomic changes in cultured keratinocytes, including disruption of cholesterol biosynthesis, that mimic changes seen in patients with AD.^16^ The predicted functional network in Fig 3 comprises upregulated and downregulated lipid metabolism genes and might offer insight into the complex interplay of metabolic dysfunction with systemic inflammation.^52^ Genes encoding proteins involved with very long-chain fatty acid CoA ligase activity are downregulated, which is in keeping with the observation that ceramides and long-chain fatty acids play an important role in skin barrier formation.^49,53,54^ Our stratified analysis indicates that therapies aimed to restore skin lipid composition might be most beneficial to *FLG* wild-type patients.

The *FLG*-stratified case-control and case-case comparisons have identified an overlapping functional network of proteins forming a type 1 interferon–mediated defense response (Fig 6). The upregulation of this network might relate to the dysfunctional cutaneous response to viral infection in patients with AD, which can be a significant problem in clinical practice.^5^ Alternatively, it might represent a suboptimal, partially functional mechanism to compensate for the increased frequency of viral infections, including eczema herpeticum,^55^ seen in filaggrin-deficient subjects. The signal transducer and activator of transcription (STAT) encoded by *STAT1* contributes to the transcriptional control of several interferon-stimulated genes, including *IFITM1*, *IFITM2*, *IFI27*, and *GBP1.* The Janus kinase/STAT signaling pathway plays a key role in transmembrane signaling from the T_H_2 cytokines IL-4 and IL-13, which predominate in acute AD.^56^ *IFITM1* and *IFITM2* encode interferon-induced transmembrane proteins, which contribute to the control of cell growth through a multimeric complex involved in the transduction of antiproliferative and homotypic adhesion signals; they are induced by IFN-γ in primary keratinocytes *in vitro* and have been proposed to play a role in keratinocyte apoptosis in patients with AD.^57^ The chemokine network encoded by genes including *CCL13, CCL18, CXCL9*, and *CXCL10* has been implicated in the pathogenesis of AD in some of the previous microarray studies.^9,11,13,58^ Filaggrin haploinsufficiency increases the risk of eczema herpeticum,^59^ and the functional network predicted by transcriptomic analysis indicates a pathway that might be targeted for therapeutic intervention in susceptible patients.

The quantitative reduction in filaggrin mRNA with *FLG* null mutations (Fig 4) is consistent with previous studies showing a stepwise reduction in filaggrin breakdown products in *FLG* heterozygotes and *FLG* homozygotes or compound heterozygotes.^27,60^ One previous microarray study has used *FLG* genotype–stratified analysis^8^ and reported no significant difference in *FLG* mRNA levels between *FLG* wild-type cases and control subjects. In our analysis comparison of all cases with all control subjects also showed no difference in *FLG* mRNA counts (*P* > .05), but we have shown a significant difference between *FLG* expression in wild-type control subjects compared with wild-type cases (Fig 4). This is compatible with the confidence range of data published previously^8^ but suggests that there might be compensatory upregulation of *FLG* expression in our pediatric AD cases. An alternative explanation is that there might be lower filaggrin expression in our control subjects, possibly reflecting the slightly older age, but detailed studies of filaggrin expression changes with aging are not currently available.

Intercellular edema (spongiosis) is a characteristic feature in patients with AD, but the underlying mechanisms are unclear. The differential expression of genes encoding proapoptotic and antiapoptotic proteins (including *IGFBP6*, *CLU*, *IFITM1*, *IFITM2*, *SELE*, *CXCL10*, *PRF1*, and *IRF1*) might contribute to the propensity to keratinocyte apoptosis that some authors consider to be a key pathomechanism in atopic spongiosis.^57,61,62^ Alternatively, the dysregulation of proteins in cell death pathways might reflect the specialized process of keratinocyte cell death, cornification.^63,64^

The majority of AD risk loci identified by using genome-wide association studies are located within intergenic regions of unknown function. Our analysis has offered insight into possible genetic mechanisms associated with 14 of the previously reported AD risk loci. We propose *cis*-eQTLs indicating a range of pathomechanisms, including structural (*FLG*, *LCE3E*, and *TNXB*), immune response (*CST6*, *HLA-DRA*, *IRF1*, and *PRRT1*), transcriptional regulation (*ATF6B*, *C11orf30*, *RP11-21L23.4*, *RPL3P2*, *RPSAP47*, *SIPA1*, and *ZNF652*), mitochondrial *(TST)*, and lipid biosynthetic *(AGPAT1)*.

The use of DRS has allowed detailed study of tissue-specific gene expression data from small amounts of tissue. This transcriptomic analysis has provided new insight into the mechanistic pathways in atopic skin, which are both dependent and independent of *FLG* genotype. The strength of using a functional genotype for phenotype stratification is apparent, and this approach might prove useful for other tissue-specific inflammatory disorders and personalized medicine in the future.

**Key messages**

• Atopic skin shows differential gene expression in pathways classified in the extracellular space, lipid metabolism, and stress response.
• Substratification of the whole transcriptome data set according to *FLG* genotype reveals a type 1 interferon–mediated stress response in filaggrin-deficient skin.
• These findings offer insight into the underlying abnormalities in uninflamed atopic skin and might guide future therapy development.

## Figures and Tables

**Fig 1:**
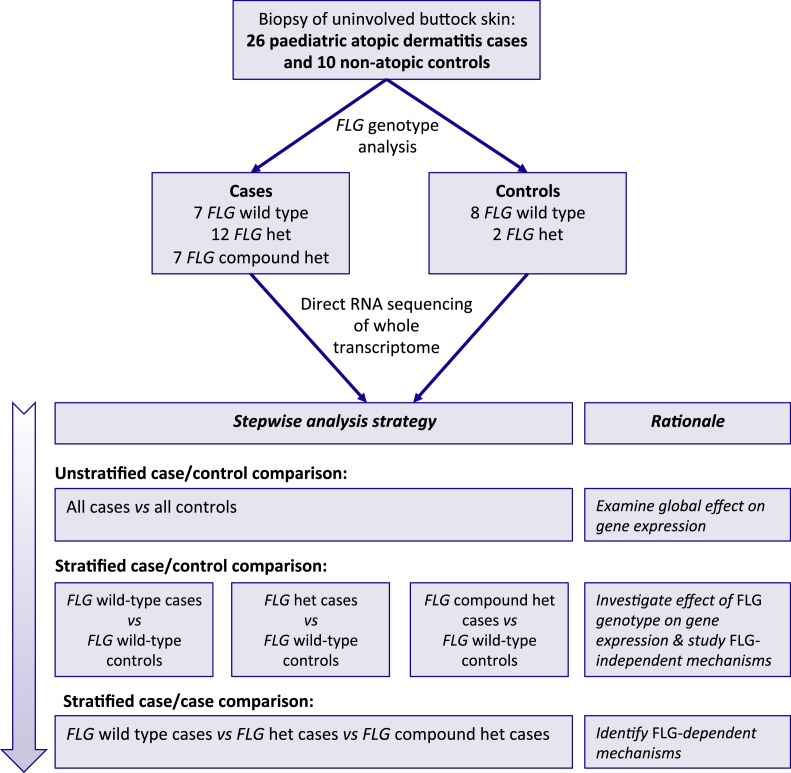
Flow diagram summarizing study design and analysis strategy. *het*, Heterozygous.

**Table I:** *FLG* genotype and demographic data for 26 pediatric patients with moderate-to-severe AD and 10 nonatopic control subjects

Phenotype	*FLG* genotype	No. (%)	Mean age (y [range; SD])	Male sex, no. (%)
Cases	Wild-type	7 (27)	12.3 (9-15; 2.8)	7 (100)
	Heterozygous	12 (46)	11.6 (6-16; 3.4)	8 (67)
	Compound heterozygous	7 (27)	10.7 (6-13; 3.4)	4 (57)
	Total	26	11.5 (6-16; 3.2)	19 (73)
Control subjects	Wild-type	8 (80)	17.9 (16-19; 0.8)	4 (50)
	Heterozygous	2 (20)	18.0 (18-18; 0.0)	2 (100)
	Compound heterozygous	0 (0)	NA	0 (0)
	Total	10	17.9 (16-19; 0.7)	6 (60)

Cases were diagnosed by experienced pediatric dermatologists (G.M.O'R., R.M.W., A.D.I., and S.J.B.), and severity was defined by using the Nottingham eczema severity score.^34^ Heterozygous mutations in the cases were p.R501X (n = 6) and c.2282del4 (n = 6), and those in the control subjects were p.R501X (n = 1) and c.5690delA (n = 1). Compound heterozygous genotypes (each n = 1) were p.R501X/c.2282del4, p.R501X/p.S3247X, p.R501X/p.G1139X, p.R501X/p.S1280X, c.2282del4/p.R1474X, p.S1040X/c.10885delC, and p.S608X/p.Y2092X. *NA*, Not applicable.

## Supplementary data

Methods

Table E1

Table E2

Table E3

Table E4

Table E5

Table E6

Table E7a

Table E7b

Table E8

Table E9

Table E10

Fig E1

Fig E2
