# Supplementary material for: Filaggrin-stratified transcriptomic analysis of pediatric skin identifies mechanistic pathways in patients with atopic dermatitis
Source: J Allergy Clin Immunol. 2014 Jul;134(1):82–91. doi: 10.1016/j.jaci.2014.04.021 (PMC4090750; doi:10.1016/j.jaci.2014.04.021)
Supplement: Methods [file mmc1.docx]

**Supplementary methods**

**Direct RNA sequencing (DRS)**

DRS technology (Helicos® Genetic Analysis System, Cambridge, MA) allows single molecules of mRNA to be sequenced directly from the 3’ end without the need for reverse transcription, shearing and amplification steps required by other RNA sequencing methods. In DRS, mRNA molecules are captured on a flow cell by hybridization of the poly-A tail and each molecule is directly sequenced, producing short reads (mean length 33-34 nucleotides). Biases that may be introduced by sample preparation steps are therefore reduced or eliminated and the read is innately stranded. The read count for each molecule is directly proportional to the quantity of mRNA, offering digital quantification of the transcriptome with a large dynamic range. The technology is also applicable to small quantities of RNA (<500ng). Full details of the DRS technology are available in published manuscripts.^E1, E2^

**Direct RNA sequencing read processing**

The raw sequence data were aligned to the human reference sequence (ensembl release 61) with the indexDPgenomic aligner available within the Helisphere package (http://sourceforge.net/projects/openhelisphere/). The parameters used were --best_only --min_norm_score 4.0 --strands both --alignment_type GL. The aligned data were then filtered with the filterAlign program (also in Helisphere) twice: once to remove multi-mapping reads and too short reads (parameters: --min_len 25 --min_score 4.0 --best_only --global_ambig all --local_ambig rand), and secondly to ensure reads are mapping uniquely (parameters: --global_ambig none). Finally, an in-house script was developed to remove reads with >4 indels and genomic positions with <2 reads as these reads are most likely to be noise.

The filtered reads were then annotated to genes as defined by ensembl (release 66) using an in-house script which queries the ensembl perl API taking into account the native strand specificity in DRS. As DRS reads have the unique feature of being found exclusively at the 3’ end of all transcripts they are exquisitely sensitive to the correct annotation of the 3’UTR. For many genes the 3’UTR annotation is truncated^E3-E5^ meaning that DRS reads may be not properly annotated to genes if they align beyond the truncated 3’UTR definition in the annotation. To compensate for this an additional 100bp were added to the 3’ ends of all gene coordinates in the Ensembl annotation.

**Differential gene expression and *FLG* correlation analyses**

Across the 36 samples, 13,162-16,953 genes were found to be expressed with a union set of 21,708 genes expressed in at least one sample. For each gene, its mean expression was determined across the three *FLG* genotypes in the case samples and correlated to *FLG*’s expression using Pearson’s method. Any genes which have an *r* close to 1 or -1 are the most the likely candidates to be co-regulated. In order to avoid genes with low counts having spurious correlations, only genes with a total mean expression across the three genotypes >100 reads were considered (n=4807).

A significance value for the correlations can be calculated. Firstly the *t* statistic can be determined for gene *i* as:

$$t_{i}=r_{i}.\sqrt{\frac{n-2}{1-{r_{i}}^{2}}}$$

where *r_i_* is the Pearson’s correlation and n is the values per gene (here, n=3) which determines the degrees of freedom (*n* – 2). Given *t_i_* and the degrees of freedom, a p-value can be calculated from the standard *t*-distribution using the ‘pt’ function in R. p-values are quoted unadjusted. All genes with a correlation p-value <0.05 and a log_2_ fold-change >0.5 or <-0.5 in the wild-type versus compound heterozygote comparison were considered for further investigation. All code is available from FigShare: http://dx.doi.org/10.6084/m9.figshare.879673.

The correlation between samples was tested by hierarchical clustering of the Pearson’s correlations between all pairwise comparisons. Clustering was performed with complete-linkage.

Case and control groups showed different age distributions, but controlling for age in comparative expression analyses was limited by the small sub-group sizes.

**Data Access**

Raw data have been deposited at European Genome-Phenome Archive (study number awaited) and is also available at polyAdb (http://www.compbio.dundee.ac.uk/polyAdb) along with the processed data which can be directly viewed in the Integrated Genome Browser^E6^ or as data tracks at www.ensembl.org.

**Expression quantitative trait analysis**

The location of each transcript showing significant differential expression was defined by the position of its 5’ end; these loci were systematically compared with previously reported single nucleotide variants associated with AD, reported in the NIH Catalogue of Published Genome-Wide Association Studies (https://www.genome.gov/gwastudies/ accessed November 2013) and with additional references.^E7, E8^ *Cis-*eQTLs were defined by a previously reported atopic dermatitis risk SNV from GWAS within 250kb of the transcript 5’ end; negative number indicates that the SNV is upstream of the gene.

**Quantitative PCR analysis**

9 genes that showed significant differential expression plus 3 genes (*DSP, RPL5* and *LCE2B*) that were highly but uniformly expressed were tested for validation of DRS data by qPCR. Normalised threshold concentration (Ct) values were compared between FLG genotype sub-groups using an unpaired t-test and a 2-tailed p<0.05 was considered to be statistically significant.

**References**

E1. Ozsolak F, Platt AR, Jones DR, Reifenberger JG, Sass LE, McInerney P, et al. Direct RNA sequencing. Nature 2009; 461:814-8.

E2. Ozsolak F, Milos PM. RNA sequencing: advances, challenges and opportunities. Nature reviews. Genetics 2011; 12:87-98.

E3. Sherstnev, A. *et al.* Direct sequencing of Arabidopsis thaliana RNA reveals patterns of cleavage and polyadenylation. *Nature structural & molecular biology* **19**, 845-52 (2012).

E4. Lin, Y. *et al.* An in-depth map of polyadenylation sites in cancer. *Nucleic acids research* **40**, 8460-71 (2012).

E5. Schurch, N. *et al.* Improved annotation of 3' untranslated regions and complex loci by combination of strand-specific Direct RNA Sequencing, RNA-seq and ESTs. *PLoS One* **Accepted for publication** (2014)

E6. Nicol, J.W., Helt, G.A., Blanchard, S.G., Jr., Raja, A. & Loraine, A.E. The Integrated Genome Browser: free software for distribution and exploration of genome-scale datasets. *Bioinformatics* **25**, 2730-1 (2009).

E7. Esparza-Gordillo J, Weidinger S, Folster-Holst R, Bauerfeind A, Ruschendorf F, Patone G, et al. A common variant on chromosome 11q13 is associated with atopic dermatitis. Nature genetics 2009; **41**:596-601.

E8. Paternoster L, Standl M, Chen CM, Ramasamy A, Bonnelykke K, Duijts L, et al. Meta-analysis of genome-wide association studies identifies three new risk loci for atopic dermatitis. Nature genetics 2012; **44**:187-92.
