# Supplementary material for: Filaggrin-stratified transcriptomic analysis of pediatric skin identifies mechanistic pathways in patients with atopic dermatitis
Source: J Allergy Clin Immunol. 2014 Jul;134(1):82–91. doi: 10.1016/j.jaci.2014.04.021 (PMC4090750; doi:10.1016/j.jaci.2014.04.021)
Supplement: Table E6 [file mmc7.docx]

| **Transcript (description)**  **and chromosome band** | **GWAS dbSNP id** | **Distance of gene 5’ end from GWAS SNP (bp)** | **Candidate gene associated with GWAS SNP locus** | **Other reported candidate genes within locus** |
| --- | --- | --- | --- | --- |
| ***LCE3E* (late cornified envelope 3E)** 1q21.3 | rs3126085 | -238,431* | *FLG* ^E8-E12^ | *HRNR* ^E9^ |
| *FLG* (filaggrin) 1q21.3 |  | 3,138*^§^ |  |  |
| ***IRF1* (interferon regulatory factor 1)** 5q31.1 | rs1295686 | 169,353* | *KIF3A, IL13* ^E12^ | *KIF3A, IL4,*  *IL13-RAD50* ^E8, E10^ |
| ***RPL3P2* (ribosomal protein L3 pseudogene 2)** 6p21.33 | rs9368677 | 24,227 ^+^ | *HLA-C* ^E12^ |  |
| ***ATF6B*** **(activating transcription factor 6 beta)** 6_mcf_hap5 | rs12153855 | -21,226 ^+^ | *TNXB* ^E8^ |  |
| *TNXB* (tenascin XB) 6_mcf_hap5 |  | -8,307 ^+^ |  |  |
| ***AGPAT1* (1-acylglycerol-3-phosphate O-acyltransferase 1)** 6_ssto_hap7 |  | 12,446 ^+^ |  |  |
| ***HLA-DRA* (major histocompatibility complex, class II, DR alpha)** 6_ssto_hap7 | rs9469099 | -98,711* | *C6orf10* ^E12^ |  |
| ***PRRT1* (proline-rich transmembrane protein 1)** 6_ssto_hap7 | rs176095 | 36,169* | *GPSM3* ^E12^ |  |
| ***RPSAP47* (ribosomal protein SA pseudogene 47)** 8q21.13 | rs7000782 | -162,955 *^+§^ | *ZBTB10* ^E10^ |  |
| ***CST6* (cystatin E/M)** 11q13.1 | rs479844 | -227,355 ^+^ | *OVOL1* ^E10^ | *OVOL1,* 11q13 ^E8, E12^ |
| ***SIPA1* (signal-induced proliferation-associated 1)** 11q13.1 | rs593982 | 107,539 ^+^ | *OVOL1* ^E12^ | *OVOL1,* 11q13 ^E8, E10^ |
| ***RP11-21L23.4.1* (antisense transcript)** 11q13.5 | rs11236809 | -147,057 *^+^ | *c11orf30* ^E12^ | *C11orf30 - LRRC32* ^E9^ |
| *C11orf30* (chromosome 11 open reading frame 30) 11q13.5 | rs7927894 | 145,349* | *c11orf30* ^E9^ | *C11orf30 - LRRC32* ^E12^ |
| *ZNF652* (zinc finger protein 652) 17q21.32-q21.33 | rs16948048 | 631 ^+^ | *ZNF652* ^E7^ |  |
| ***TPD52L2* (tumor protein D52-like 2)** 20q13.33 | rs909341 | -167,854 *^+^ | *TNFRSF6B* ^E7^ | *TNFRSF6B,*  *ZGPAT* ^E10, E11^ |
| ***TST* (thiosulphate sulphurtransferase)** 22q12.3 | rs4821544 | -157,178 ^+^ | *NCFA* ^E10^ |  |

**Table E6. Candidate transcripts identified as *cis-*eQTL from previous genome-wide association studies**

*Cis-*eQTLs are defined by a previously reported atopic dermatitis risk SNP from GWAS within 250kb of the transcript 5’ end; negative number indicates that the SNP is upstream of the gene. GWAS top hit SNPs are as listed in the NIH Catalogue of Published Genome-Wide Association Studies (https://www.genome.gov/gwastudies/) with additional references.^E10, E11^ Chromosome bands identified from UCSC Genome Browser (https://www.genome.ucsc.edu/cgi-bin/hgGateway) Feb 2009 (GRCg37/hg19) assembly, accessed 23 October 2013. *Indicates significantly differentially expressed transcript (FDR<0.05) in *FLG* wild-type control *versus* *FLG* compound heterozygous cases; ^+^indicates significantly differentially expressed transcript in *FLG* wild-type control *versus* *FLG* heterozygous cases; ^§^indicates significantly differentially expressed transcript in *FLG* wild-type control *versus* *FLG* wild-type cases; previously unreported *cis*-eQTL transcripts are marked in **bold** text.

***References***

E7. Ellinghaus D, Baurecht H, Esparza-Gordillo J, Rodriguez E, Matanovic A, Marenholz I, et al. High-density genotyping study identifies four new susceptibility loci for atopic dermatitis. Nature genetics 2013; 45:808-12.

E8. Weidinger S, Willis-Owen SA, Kamatani Y, Baurecht H, Morar N, Liang L, et al. A genome-wide association study of atopic dermatitis identifies loci with overlapping effects on asthma and psoriasis. Human molecular genetics 2013; 22:4841-56.

E9. Esparza-Gordillo J, Weidinger S, Folster-Holst R, Bauerfeind A, Ruschendorf F, Patone G, et al. A common variant on chromosome 11q13 is associated with atopic dermatitis. Nature genetics 2009; 41:596-601.

E10. Paternoster L, Standl M, Chen CM, Ramasamy A, Bonnelykke K, Duijts L, et al. Meta-analysis of genome-wide association studies identifies three new risk loci for atopic dermatitis. Nature genetics 2012; 44:187-92.

E11. Sun LD, Xiao FL, Li Y, Zhou WM, Tang HY, Tang XF, et al. Genome-wide association study identifies two new susceptibility loci for atopic dermatitis in the Chinese Han population. Nature genetics 2011; 43:690-4.

E12. Hirota T, Takahashi A, Kubo M, Tsunoda T, Tomita K, Sakashita M, et al. Genome-wide association study identifies eight new susceptibility loci for atopic dermatitis in the Japanese population. Nature genetics 2012; 44:1222-6.
