# Supplementary material for: Filaggrin-stratified transcriptomic analysis of pediatric skin identifies mechanistic pathways in patients with atopic dermatitis
Source: J Allergy Clin Immunol. 2014 Jul;134(1):82–91. doi: 10.1016/j.jaci.2014.04.021 (PMC4090750; doi:10.1016/j.jaci.2014.04.021)
Supplement: Table E8 [file mmc9.docx]

|  | ***FLG* wild type cases n=6^§^**  **mean Ct (SD)** | ***FLG* heterozygous cases n=12**  **mean Ct (SD)** | ***FLG* compound heterozygous cases**  **n=7 mean Ct (SD)** | | **2-tailed t-test**  **p value** |
| --- | --- | --- | --- | --- | --- |
| ***Transcripts down-regulated in DRS analysis*** | | | |  |  |
| *FLG* | 20.14 (0.72) | 21.41 (1.22) | 22.48 (0.51) | | 9.51E-14 |
| *CILP* | 27.03 (1.14) | 28.31 (1.08) | 28.38 (0.79) | | 0.00017 |
| *IGFBP6* | 27.24 (0.73) | 27.82 (1.29) | 28.15 (0.55) | | 0.00008 |
| *CLU* | 24.80 (0.52) | 25.21 (1.18) | 25.37 (0.40) | | 0.00041 |
| *C1QTNF1* | 27.18 (0.60) | 28.09 (1.15) | 27.65 (0.26) | | 0.00222 |
| *S100P* | 28.15 (0.96) | 28.98 (0.85) | 29.02 (0.96) | | 0.00347 |
| *C16orf45* | 27.69 (0.79) | 28.49 (1.04) | 28.31 (0.75) | | 0.01983 |
| *S100A9* | 25.71 (2.52) | 26.55 (2.13) | 25.27 (1.65) | | 0.53673 |
| ***Transcript up-regulated in DRS analysis*** | | | |  |  |
| *AC105404.4.1* | 35.63 (0.81)* | 35.85 (1.43)** | 35.38 (0.62)* | | 0.24629 |
| ***Transcripts unchanged in DRS analysis*** | | | |  |  |
| *RPL5* | 21.18 (0.43) | 20.89 (2.40) | 21.36 (0.63) | | 0.33916 |
| *DSP* | 19.88 (0.55) | 20.33 (0.92) | 19.74 (0.49) | | 0.38862 |
| *LCE2B* | 23.26 (0.74) | 23.81 (1.24) | 23.67 (0.54) | | 0.06047 |

**Supplementary Table 7. Results of qPCR to validate selected transcripts from direct RNA sequencing analysis**

Each experiment was performed in triplicate; Ct, threshold cycle normalised to *GAPDH*; DRS, direct RNA sequencing; SD, standard deviation; ^§^one wild-type sample failed qPCR analysis; *n=5 and **n=10 results available; 2-tailed t-test compares *FLG* wild-type cases with *FLG* compound heterozygous cases.
