# Supplementary material for: Filaggrin-stratified transcriptomic analysis of pediatric skin identifies mechanistic pathways in patients with atopic dermatitis
Source: J Allergy Clin Immunol. 2014 Jul;134(1):82–91. doi: 10.1016/j.jaci.2014.04.021 (PMC4090750; doi:10.1016/j.jaci.2014.04.021)
Supplement: Table E10 [file mmc11.docx]

|  |  | **Normalised read counts (standard deviation) for cases with different *FLG* genotypes** | | |  | | |
| --- | --- | --- | --- | --- | --- | --- | --- |
| **Gene** | **Description** | **Wild type** | **Heterozygous** | **Compound heterozygous** | **Correlation with *FLG*** | **Fold change** | **P value** |
| ***TRIM22*** | tripartite motif containing 22 | 27.8 (4.7) | 39.1 (51.6) | 47.1 (62.5) | -1.000 | 1.69 | 0.005230 |
| ***KCNK1*** | potassium channel, subfamily K, member 1 | 29.8 (7.0) | 37.4 (9.2) | 43.7 (6.7) | -1.000 | 1.47 | 0.008800 |
| ***PARP9*** | poly (ADP-ribose) polymerase family, member 9 | 31.4 (12.0) | 40.2 (45.5) | 48.3 (53.5) | -0.998 | 1.54 | 0.019400 |
| ***IFITM2*** | interferon induced transmembrane protein 2 (1-8D) | 161.0 (33.5) | 220.0 (226.0) | 248.0 (261.0) | -0.993 | 1.54 | 0.037500 |
| ***IFITM1*** | interferon induced transmembrane protein 1 | 142.0 (35.6) | 202.0 (226.0) | 230.0 (251.0) | -0.992 | 1.61 | 0.039900 |
| ***STAT1*** | signal transducer and activator of transcription 1 | 59.5 (7.6) | 112.0 (188.0) | 134.0 (185.0) | -0.989 | 2.26 | 0.048200 |
| ***NOV*** | nephroblastoma overexpressed gene | 52.6 (16.4) | 45.7 (14.2) | 37.0 (8.9) | 0.988 | 0.70 | 0.048700 |
| ***PIP*** | prolactin-induced protein | 513.0 (529.0) | 283.0 (243.0) | 188.0 (186.0) | 0.989 | 0.37 | 0.047800 |
| ***MT-ND1*** | mitochondrially encoded NADH dehydrogenase 1 | 364.0 (91.6) | 310.0 (90.9) | 245.0 (40.5) | 0.990 | 0.67 | 0.046000 |
| ***IGFBP6*** | insulin-like growth factor binding protein 6 | 84.2 (40.3) | 65.5 (20.7) | 44.9 (8.3) | 0.994 | 0.53 | 0.036200 |
| ***C13orf15*** | chromosome 13 open reading frame 15 | 73.6 (28.5) | 58.3 (25.0) | 42.3 (10.9) | 0.995 | 0.58 | 0.032200 |
| ***C15orf48*** | chromosome 15 open reading frame 48 | 107.0 (64.6) | 73.2 (18.9) | 55.5 (22.6) | 0.996 | 0.52 | 0.029100 |
| ***MT-CO1*** | mitochondrially encoded cytochrome c oxidase I | 461.0 (118.0) | 371.0 (138.0) | 281.0 (58.5) | 0.996 | 0.61 | 0.027800 |
| ***KLF9*** | Kruppel-like factor 9 | 60.4 (35.5) | 49.1 (18.3) | 38.6 (12.2) | 0.998 | 0.64 | 0.020500 |
| ***RNF152*** | ring finger protein 152 | 43.0 (7.6) | 36.1 (8.9) | 29.8 (6.9) | 0.998 | 0.69 | 0.019700 |
| ***COL12A1*** | collagen, type XII, alpha 1 | 78.4 (34.7) | 62.2 (15.6) | 52.6 (24.3) | 0.998 | 0.67 | 0.019700 |
| ***CA2*** | carbonic anhydrase II | 64.8 (58.9) | 49.0 (16.1) | 39.5 (12.2) | 0.998 | 0.61 | 0.018000 |
| ***S100P*** | S100 calcium binding protein P | 62.4 (18.1) | 47.2 (16.6) | 33.6 (23.7) | 0.999 | 0.54 | 0.017300 |
| ***MUCL1*** | mucin-like 1 | 463.0 (293.0) | 347.0 (195.0) | 245.0 (204.0) | 0.999 | 0.53 | 0.015500 |
| ***TPM2*** | tropomyosin 2 (beta) | 107.0 (65.9) | 89.8 (40.5) | 74.5 (21.3) | 0.999 | 0.70 | 0.015500 |
| ***SLC12A2*** | solute carrier family 12, member 2 | 218.0 (78.8) | 179.0 (70.9) | 154.0 (49.5) | 0.999 | 0.71 | 0.012400 |
| ***PRR4*** | proline rich 4 (lacrimal) | 53.6 (59.6) | 39.8 (68.8) | 30.2 (24.1) | 1.000 | 0.56 | 0.005810 |
| ***SCGB1D2*** | secretoglobin, family 1D, member 2 | 106.0 (62.8) | 61.0 (64.8) | 29.6 (16.9) | 1.000 | 0.28 | 0.004970 |
| ***PRELP*** | proline/arginine-rich end leucine-rich repeat protein | 60.3 (23.3) | 49.0 (8.1) | 40.5 (10.9) | 1.000 | 0.67 | 0.002350 |
| ***ZG16B*** | zymogen granule protein 16 homolog B (rat) | 49.0 (32.4) | 38.4 (14.5) | 30.4 (11.7) | 1.000 | 0.62 | 0.000845 |
| ***FLG*** | filaggrin | 3290.0 (677.0) | 1930.0 (459.0) | 917.0 (270.0) | 1.000 | 0.28 | 4.74E-09 |
