# Supplementary figures and images for: Filaggrin-stratified transcriptomic analysis of pediatric skin identifies mechanistic pathways in patients with atopic dermatitis
Source: J Allergy Clin Immunol. 2014 Jul;134(1):82–91. doi: 10.1016/j.jaci.2014.04.021 (PMC4090750; doi:10.1016/j.jaci.2014.04.021)

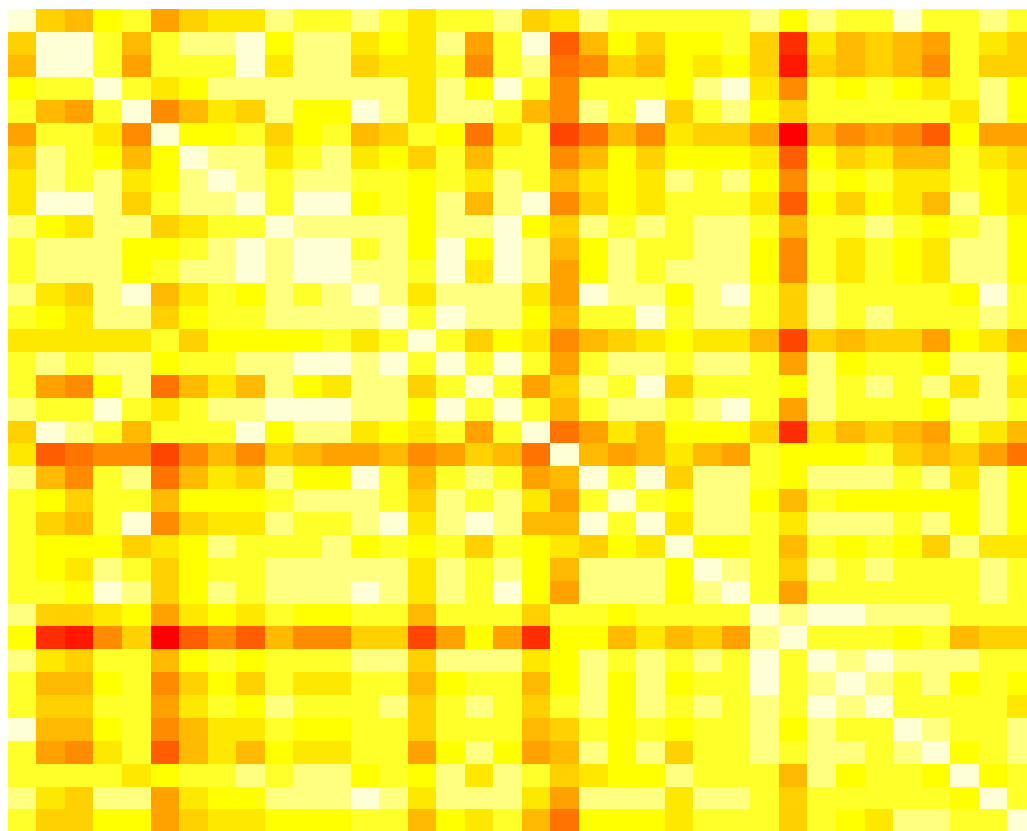[illegible]

Supplement: Fig E1 [file mmc12.pdf]

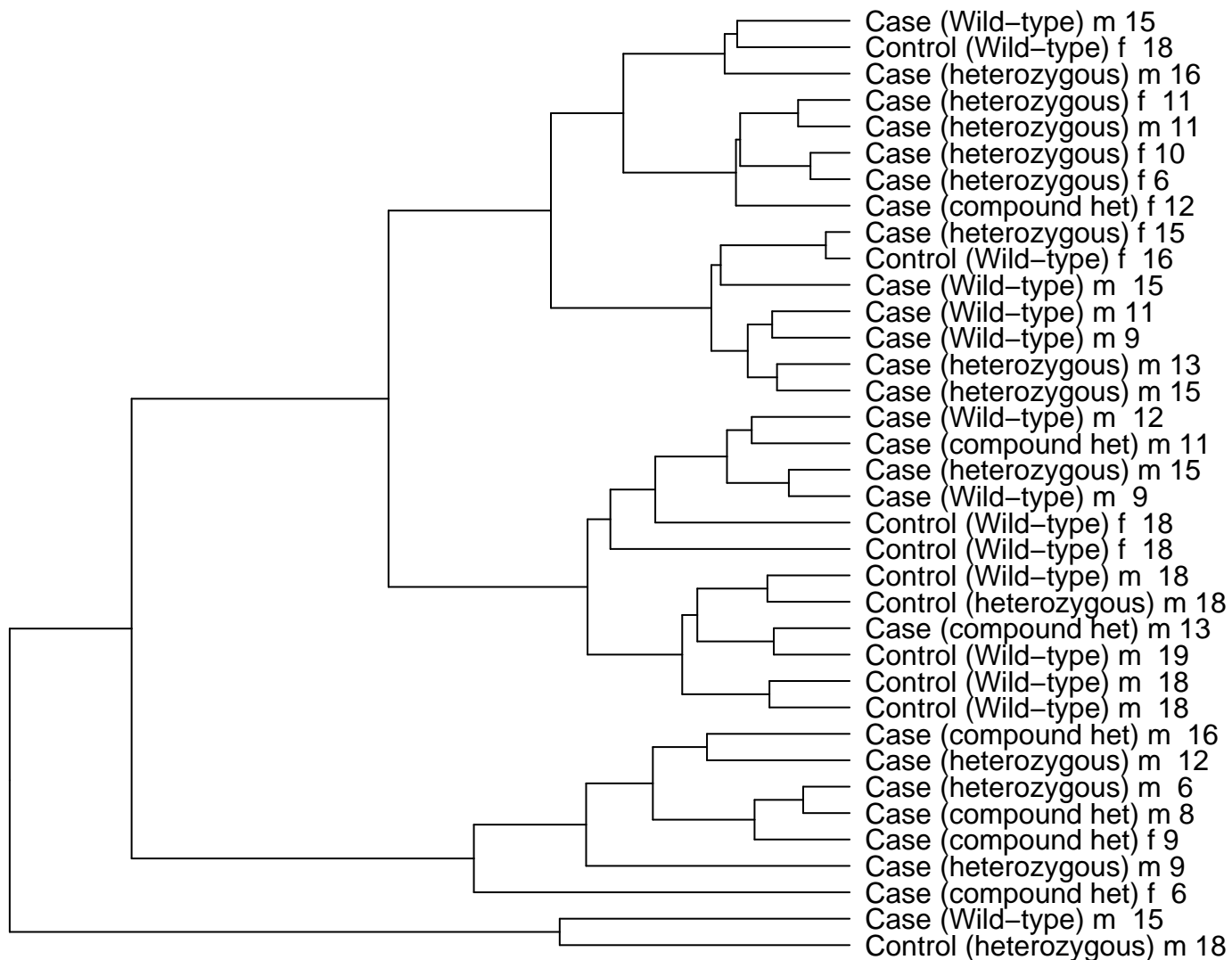

Supplement: Fig E2 [file mmc13.pdf]
